# “There is people like us and there is people like them, and we are not like them.” Understating social exclusion – a qualitative study

**DOI:** 10.1371/journal.pone.0253575

**Published:** 2021-06-22

**Authors:** Patrick O’Donnell, Lisa Moran, Stefan Geelen, Diarmuid O’Donovan, Maria van den Muijsenbergh, Khalifa Elmusharaf

**Affiliations:** 1 School of Medicine, University of Limerick, Limerick, Ireland; 2 Health Research Institute, University of Limerick, Limerick, Ireland; 3 Radboud University Medical Centre, Department of Primary and Community Care, Nijmegen, The Netherlands; 4 Centre for Public Health, School of Medicine, Dentistry and Biomedical Sciences, Queens University, Belfast, Northern Ireland; 5 Pharos, Dutch Centre of Expertise on Health Disparities, Utrecht, The Netherlands; University of Perugia, ITALY

## Abstract

Social exclusion is a complex concept that is relevant in terms of the health of vulnerable groups. Attempts have been made in the past to measure it, both at the population and the individual level. The aim of this research was to engage with a broad range of relevant stakeholders in Ireland in order to learn how they defined and conceptualised social exclusion. Semi-structured interviews were carried out with 24 participants selected using maximum variation sampling. One quarter of the interviewees were experts by experience. Participants included academic experts, the heads of organisations working nationally with socially excluded groups, politicians, clinicians, support workers and health service managers all with experience of working with socially excluded groups. The resulting definition of social exclusion was “the experience of lack of opportunity, or the inability to make use of available opportunities, thereby preventing full participation in society.” From this, we developed a new model of the concept comprising three elements; Opportunities, Influencing factors and Social outcomes. Opportunities are the fundamental needs that are required to be met for a person to begin leaving social exclusion. Influencing factors are a mixture of the personal characteristics and more complex problems such as the intergenerational effects of disadvantage. Social outcomes include a person being accepted by wider society, and subsequently being able to participate. The conceptual framework we developed can contribute to a better understanding of the concept of social exclusion. The traditional policy focus on improving the needs of excluded people at the Opportunities level must continue, but must be complemented by tackling the problems at the levels of the Influencing factors and Social outcomes also. In terms of changes to practice, the measurement of the social exclusion status of people engaging with primary care and other services would be an important start in order to better understand the magnitude of the work required.

## Introduction

Social exclusion is a concept that has risen to prominence in recent years, and it has been recognised as a major contributing factor to the poor health and early death of the people affected by it [1–4]. The adoption and utilisation of the term by European Union bodies, the World Bank and the World Health Organization (WHO) has cemented its relevance in discussion on the provision of care for groups of people who are considered to be marginalised or vulnerable [5–8]. These groups include people who are homeless, prisoners, sex workers and individuals with substance use disorders [2,9]. The seminal WHO Social Exclusion Knowledge Network report of 2008 described people being socially excluded as a result of a combination of disparities in access to resources, differences in the capabilities of individuals to use those resources, and inadequate rights and legal freedoms [4]. An important feature of the concept of social exclusion is that it is concerned not just with a person’s income and risk of poverty; but that it also incorporates political, social and cultural facets of their lives [10]. The WHO have said that people affected by social exclusion should be involved in the development of policies and services relevant to their needs [10]. Social inclusion and social exclusion are often described as being on a continuum, and researchers have sought to develop measurement tools in order to try and discover where an individual is positioned on that spectrum [11,12]. There have also been a number of conceptual and literature reviews published on the topic of social exclusion [8,13–16]. Despite the frequent use of the term social exclusion by scholars, planners and practitioners in the domain of health, lack of clarity on its components means that it remains an underutilised concept in terms of attempting to improve the health of society’s most marginalised people. This was reflected in the findings of a recent mapping of the Irish context that we conducted [17]. We also discovered there was subsequent confusion on who was responsible for implementing actions designed to mitigate social exclusion.

Over the years, there have been numerous attempts made to measure social exclusion at both the individual and population levels, and this has resulted in the development of a number of tools [11,12,18]. A scoping review we conducted on this topic showed that the vast majority of the tools for the assessment of individuals were designed for use in mental health settings, and none of them specifically mentioned primary healthcare as a location for the assessment of social exclusion [19]. Only a very small number of the 22 measurement tools analysed in our review were created with marginalised groups other than mental health patients in mind. There was also an obvious lack of consensus on a working definition of social exclusion employed by the researchers developing the measurement tools [19]. In recent years there has been a particular focus on the health of people who are excluded in society, and this targeted focus of activity has been named ‘inclusion health’ [1,20]. The overarching goal of the inclusion health agenda is to promote research, policy and clinical initiatives that seek to meet the crosscutting needs of the most excluded members of society. To do this effectively, the voices of those who are affected by exclusion should be included in the development of relevant plans [20,21].

There is a separate body of literature that has been published on the assessment of physiological or biochemical responses to social exclusion [22–25]. Many of these papers have examined the responses of animal or human participants to simulated exclusion in real world or laboratory settings. The working definition of social exclusion used by many of the researchers on these projects is often narrow compared to that used by authors in the sociological or medical literature, for example Benenson et al. [26] describe social exclusion as “an episode in which same-sex friends or close acquaintances took part in a joint activity without the participant, in a situation where the participant was available and would have expected to be included.” For the research we propose, we are concerned more with the experiences and perceptions of our participants rather than their physiological responses to exclusion or marginalisation.

Therefore, the aim of this research was to engage with experts to capture their understanding of the concept of social exclusion in the Irish context. These experts would have knowledge of social exclusion–either through personal experience or through their work, and have an understanding of the links between health and social exclusion. The objectives were to create a definition of social exclusion, to analyse the domains underlying the construct and to develop a framework for clearly explaining the construct. In future, this may allow us to develop a measurement tool informed by their views on the concept.

## Methods

### Study context

This research was carried out in the Republic of Ireland. There is no universal entitlement to free healthcare in Ireland; the system has ‘two-tiers’ with eligibility decided based on income thresholds [27]. Health policy is formulated by the Department of Health, and the Health Service Executive (HSE) then works to operationalise those policies [17]. One division of the HSE is concerned with Social Inclusion, and its remit is to ensure that the needs of marginalised groups are met in their engagements with health services. HSE staff do some of the work of the SI division, but staff from Civil Society Organisations (CSOs) carry out the majority. Examples of services provided by these CSOs include healthcare for people who are homeless and refugees.

### Sample selection procedure

We utilised maximum variation sampling when selecting our participants [28]. This allowed us to engage with an array of stakeholders linked to social exclusion (see Table 1). We recognised that it would not be possible to interview participants linked to all socially excluded groups, so we based our decision on groups mentioned in published literature [2,9,17,20]. These ‘inclusion health’ target groups included people who were homeless, people with addiction issues, sex-workers, people who had a history of imprisonment and members of the Irish Traveller population. It is recognised that many in these groups have significant challenges in terms of their health, and consequently have very poor morbidity and mortality statistics [2,20,29]. Access to potential participants was gained using the combination of an informal network of healthcare professionals working on social exclusion, and direct approaches to people working nationally on social exclusion. For interactions with potential participants who were considered experts by experience (EBE), we used gatekeepers from the relevant CSOs to guide us [30,31]. The 24 participants were drawn from a variety of backgrounds, with some identifying themselves as being in two stakeholder categories. Participants were chosen based on their experience or the nature of their work. Participants from the expert by experience group were suggested by knowledgeable gatekeepers as they fell into two or more of the inclusion health groups of interest and had significant experience of being socially excluded. Other participants included academics who were national experts in topics relevant to inclusion health groups, the heads of CSOs working nationally with inclusion health groups or on social exclusion more widely, the heads of health service departments concerned with inclusion health groups, a national politician with experience of working with and advocating for inclusion health groups, clinicians and support workers with more than 10 years’ experience of working with inclusion health groups, health service managers working with inclusion health groups and a manager from a philanthropic organisation that focused on inclusion health groups and social exclusion more widely. Theoretical data saturation was reached, and interviews ceased at number twenty-four.

**Table 1:** Details of participant engagement.

Participant number	Interview length (minutes)	Participant category
1	78	CSO
2	68	EBE
3	63	EBE
4	79	CSO
5	61	CSO & EBE
6	37	Prov
7	49	Prov
8	75	Prov
9	50	CSO
10	45	Pol
11	77	Acad & EBE
12	34	Prov
13	51	Prov & Pol
14	50	Aca
15	73	Prov
16	69	Pol & EBE
17	38	Acad & Prov
18	45	Phil
19	55	Acad & Prov
20	64	CSO & Pol
21	57	Acad & Prov
22	28	Acad & Pol
23	28	Prov & Pol
24	32	EBE

Key: CSO–civil society organisation, EBE–expert by experience, Prov–service provider, Pol–policymaker or politician, Acad–academic, Phil–philanthropic organisation.

### Ethical considerations

The topic of social exclusion is a delicate one, and this research sought to analyse it in a sensitive way, particularly when engaging with EBE. Experienced staff from CSOs known to these participants acted as gatekeepers and provided support. Any agreement that an EBE would be interviewed could be withdrawn at any stage if circumstances changed. The lead researcher was a general practitioner and researcher who had worked extensively with socially excluded people; this meant that he knew some of the participants (including EBE) prior to this research. This was a factor that was considered at all stages of the research, and actions were taken to mitigate any potential adverse consequences of this [31,32]. Reflective notes were also made after each interview in order to document any ethical challenges that arose. Ethical approval was granted by the Irish College of General Practitioners Research Ethics Committee. Full-informed written consent was documented with each participant.

### Data generation

This research used qualitative research methods, and we adhered to the interpretive paradigm throughout. Semi-structured interviews were carried out using a topic guide based on the literature and the experiences of the research team (Appendix 1 in S1 File). It was flexible, for example, we added the question on whether social exclusion could occur without income poverty after a detailed discussion on this point with an early participant. Based on reviews of the extant literature and our early qualitative analysis on the data for this research, we had also developed a description of social exclusion, and this was discussed with each interview participant [17,19,31]. Interviews were digitally recorded and professionally transcribed. Participants were offered the chance to review their transcripts. The Consolidated criteria for reporting qualitative studies (COREQ) was also used as a guide [33].

### Data analysis

Thematic analysis was carried out broadly following the steps described by Braun and Clarke [34]. Early analysis began during the data collection phase, and this reinforced the iterative nature of this research. After analysis of the first four interviews, an early conceptual model of exclusion was developed. Each researcher then focused on a different aspect of that model when analysing all 24 transcripts. Nvivo 12 was used for coding. Regular data analysis meetings were held for discussion of any issues arising. Two larger data synthesis workshops were held to finalise the analysis.

## Results

We will now report the results of our research initially looking at our participants definitions of social exclusion and what it encapsulates. We will then explore the fundamental Opportunities and the Influencing Factors that moderate the journey of a person beginning to move on from exclusion. Finally, we will look at the Social Outcomes and potential results of leaving exclusion.

### Defining social exclusion

#### Complexity

Four participants began by commenting on the difficulty of trying to succinctly explain the concept of social exclusion. Also, the idea that a definition of social exclusion may change depending on who was using the term was alluded to:

*Its fluid*. *There are many different terms* … *and a lot of people own the term social exclusion*. *P13 (Prov & Pol)*.

Another participant who was involved in frontline work with people who were excluded felt it was something that was relatively easy to recognise in a person, but that it was hard to actually explain the term; “It’s definitely ‘you know it when you see it’” P17 (Acad & Prov). When participants began to elaborate on their understanding of the term social exclusion, patterns began to emerge. Ten participants described the groups that they felt were affected by social exclusion; some referenced a single group, while others referenced multiple vulnerable groups:

*Those people that wouldn’t normally sit within any particular category*. *So you are talking about people like [Irish] Travellers*, *you are talking about homeless people*, *you are talking about any ethnic minorities … I’d say that would kind be the group I would certainly feel that are excluded*. *P6 (Prov)*.

#### A multidimensional concept or merely a lack of money?

Almost all interviewees explained that there were many elements that made up this concept. Seven focused on resources such as education, housing and healthcare, and referred to these as the “normal things that we can access” P12 (Prov), or “the basic services that other people can access” P11 (EBE & Acad). The implication of these comments was that these were fundamental considerations that should be available to all.

*Social exclusion is the of experience of not having access to valuable resources whether those are social*, *or material*, *or economic*, *that citizens generally have a right to*, *and access to*. *P14 (Acad)*.

One participant, P10 (Pol), viewed social exclusion as heavily weighted towards one basic resource only–income and material resources. Two other participants mentioned income and a lack of money as their first points when asked to define social exclusion.

*My version of social exclusion is linked to material resources … I am not in favour of this idea of social exclusion can be whatever you want to make it up yourself*, *and it’s disentangled from material resources*. *That’s the core of it*. *P10 (Pol)*.

Four of the participants also included reference to ‘normal’ or ‘mainstream’ society when they spoke. P7 (Prov) explained what she meant by the word mainstream:

*Mainstream I suppose is where most people fit along*, *there is a certain course*, *you know … most people are born in to a fairly decent family*, *you go to school*, *you get educated*, *you get a job*, *and they cycle starts again*, *you get your own life*, *where you’ve got enough money*, *enough education and enough health to be able to have a decent quality of life*. *And that if you do need things*, *that you know how to access them*, *and they are easy for you to access*. *P7 (Prov)*.

Many of the descriptions of the concept of social exclusion referred to agents that were likely to have intentionally or unintentionally caused harm, and led to exclusion. P16 (EBE & Pol) summarised this well when she said “To be socially excluded, it sounds like something that’s done to somebody”. Nine interviewees felt that people in wider society were at fault. One said that their experience of social exclusion was linked with their perception of how other people viewed them:

*Social exclusion*, *I would think of stigmas [sic]*, *do you know*, *like stigmas*. *When I say stigmas I mean things like*, *because I am in [named homeless hostel] so I must be a whore [sex worker]*, *or there is something wrong with me*, *or I must be a drug addict*, *do you know what I mean*? *P24 (EBE)*.

Other participants blamed societal structures or rules that are imposed; “It’s totally structural” P17 (Acad & Prov), meaning that society can often be set up to exclude people who are felt to be different or challenging. The way that services were designed to cater for the needs of the ‘mainstream’ was often seen as neglecting the needs of vulnerable people:

*There are a group of people in society who are excluded from accessing the basic services that other people can access*, *like education and health*, *and not because those services aren’t available to them*, *it’s because the services that exist are not designed in a way that they can meet the needs of the people who need them the most*. *P11 (EBE & Acad)*.

As a result of the societal structures and barriers imposed, individuals or groups that were attempting to find their way out of exclusion were sometimes led to believe that it was their own individual failings or behaviours that had led to their exclusion, and that there was little point in them trying to overcome the obstacles that they faced:

*When someone has been socially excluded for a long time*, *they feel the sense of*, *this idea that they are supposed to be the ones that are able to overcome that social exclusion*, *that it’s somehow personal to them*, *and it’s not actually a systematic*, *community wide*, *society wide epidemic … then there is no real hope I suppose of being able to challenge those structures and change them [sic] structures because people are so inward focussed on their own failures*. *P16 (EBE & Pol)*.

### Opportunities

Opportunities are the fundamental needs that are required to be met for a person to begin leaving social exclusion. These include finance, education, housing, employment and healthcare. Participants used a variety of terms to describe these needs; including “conditions”, “resources”, “rights”, “social structures” and “circumstances”. Participants felt that the onus was primarily on the State to provide access to a minimum level of these resources e.g. a minimum level of income or basic accommodation. To illustrate their points, the participants generally used examples of these five basic resources being denied, or being provided in limited ways, to people who were socially excluded.

Decision-makers and people who designed services that provided access to these fundamental entitlements were felt to be detached from the challenges people faced. If a service or resource was provided but not utilised, then negative assumptions were often made about people’s interest and willingness to engage.

*If a woman doesn’t turn up for an appointment because she has chosen to go to work*, *because if she doesn’t she won’t be able to pay for whatever that week*, *there is very little ability to understand*, *and they’ll say they are not motivated*, *and all this kind of stuff*, *whereas actually there is very little insight in to what it is like to live hand-to-mouth*. *P11 (EBE & Acad)*.

Often the structure of these services, their perceived inflexibility and complexity, seemed to discourage people who were socially excluded from applying for them. Also, the attitudes of some staff working at these services–people who were essentially controlling access to these fundamental resources–were described as being discouraging by one interviewee.

#### Finance

Participants acknowledged that the provision of a basic level of income by the State was very important. It was noted, however, that the media and others in the ‘mainstream’ were sometimes critical of people who availed of payments. It was often implied in some disparaging narratives that people did not deserve the supports. These same negative undertones were seen in descriptions of those availing of basic housing provided by the State.

*You will read articles where they talk about people sponging off the state*, *so you are ashamed for the times that you do access something that’s available to you*, *like some funding*, *or like some support when you move in to a new home and stuff*, *but then you are also put down in shame for it*. *So when you do access your rights you are often shamed for actually accessing them in the first place*. *P16 (EBE & Pol)*.

Accepting supports and being proactive in accessing opportunities that a socially excluded person was fully entitled to was often another reason that they were stigmatised and criticised.

#### Education

The importance of education as a foundation on which to build other opportunities such as employment was noted. While the State provided free basic education, not everyone could avail of it equally.

*They are entitled to free education*, *but unless extra work is done to keep them in the school and to encourage them to go to school*, *then that goal is never going to be achieved*. *And then if you add other things to the mix like children ending up in care [of the State]*, *and disturbed childhoods moving [from] place-to-place*, *or people from the Travelling community*, *you can see quite simply on paper—yes they are entitled to education*, *but in reality it’s not what happens*. *P1 (CSO)*.

#### Housing

As P14 (Acad) said, being without “a home of one’s own, yeah that would be one of the most fundamental forms of social exclusion”. This sentiment was echoed in other interviews, and while there were specific challenges relating to certain groups of people, most participants agreed that having housing was a key starting point for making progress. P15 (Prov) gave the example of the Irish Traveller community and their struggles with accommodation issues:

*They would feel isolated from their own community and living amongst settled population can be difficult if you don’t have family support nearby*, *because they do rely very much on the extended family network*, *so that would be one aspect*. *Second aspect is if you are in a community where people don’t like Travellers*, *and where they are quite discriminatory against you*, *it can be a very negative experience*,. *P15 (Prov)*.

#### Employment

Many interview participants felt that regular work unlikely to be initially possible for the excluded people they knew. The challenges of finding and maintaining a suitable job were immense, and often required some other important issues to be addressed first, as P12 (Prov) highlighted here:

*They have to start small*, *there is no point in some of our clients being offered a full time job somewhere tomorrow or Monday*, *they are not able*. *You know*, *you have to start them off with little courses and things along the way*. *P12 (Prov)*.

Unsurprisingly then, the importance of having tailored training and supported employment schemes available for people who were seeking to move from exclusion was highlighted by three interviewees:

*When I was an addict I was down town tapping [begging]*, *and I know that’s the furthest thing away from normal society as you would ever put [sic]*, *but I turned all that around and went away got a CE [community employment] scheme*, *started volunteering first*, *then I got the CE scheme*, *now I am working four and a half days a week*. *I know myself its doing good for me*. *P2 (EBE)*.

#### Healthcare

Accessing appropriate healthcare was acknowledged as being very important for people who were socially excluded. P22 (Acad & Pol) highlighted this by saying that in her opinion health “knowledge and awareness and education diminish social exclusion.” Socially excluded people often had a much greater need for health services than those who were not excluded, and this was seen as a logical reason to provide extra supports. There was frustration with the approach that some services had decided to adopt, maintaining that treating everybody equally irrespective of specific needs was what they were required to do:

*A lot of services will say to us*, *both within the health service and in the community and voluntary sector*, *will say to us look we treat everyone the same here*, *and what we try and say is well in doing that you are actually perpetuating the inequality that exists there*, *you have to recognise the difference here*. *P15 (Prov)*.

Unfortunately, the experiences of some excluded people when they did try to engage with health services were not positive or encouraging, and eight of the interviewees highlighted this. Here P17 (Acad & Prov) described the health system itself as being damaging:

*First of all when you are in a health system you are very vulnerable*, *you know*, *you are really vulnerable in so many ways … So it’s somewhere where you have to be where people can really judge you*, *where you can’t not be around the people who are going to judge you*, *or look at you that way*. *I think it’s a huge*, *huge perpetuator of structural violence*. *P17 (Acad & Prov)*.

Positive attitudes of healthcare professionals was also mentioned as being crucial in order to increase the likelihood of excluded people engaging, and maintaining that engagement, so they could address their health needs. Primary care was cited as a setting where much of that contact happened, and so it was felt to be particularly important for professionals in that space to be acutely aware of the needs and challenges faced by people who were socially excluded.

### Influencing factors

The components we will discuss in this section were seen as influences that could affect whether a person who was socially excluded could effectively combine and utilise the opportunities available to them in order to begin to leave exclusion.

#### Intergenerationality

The influence of adverse life and social circumstances across generations of families was mentioned by three participants. The impact these had on the lives of socially excluded people could not be underestimated, but these influences were sometimes forgotten:

*I think people blame themselves for their own situations*, *but society also blames people for their own situations … the second they become parents they stop being viewed as people that were socially excluded*, *and that were living in their own poverty*, *and living in their addiction … society kind of places the role of breaking that intergenerational social exclusion on to them*, *you know*, *as if somehow they weren’t also entrenched in that same poverty from the beginning*, *exactly*, *or from their own parents and the grandparents*. *P16 (EBE & Pol)*.

Conversely, the stability and resources that were usually easily available to many non-excluded people were seen as protective factors:

*Most people are born in to a fairly decent family*, *you go to school*, *you get educated*, *you get a job*, *and they cycle starts again*. *P7 (Prov)*.

#### Agency

The term agency described a person’s ability to make positive things happen for themselves: a person’s capacity to effectively coordinate, plan and carry out activities that may improve their lives. P8 (Prov) described it as:

*A learned skill*, *and I suppose it’s around being able to negotiate your way round something*, *and that would be an aspect of agency*, *I think*, *that you are able to do that kind of negotiation and bargaining piece*. *Some of it is with the sense of entitlement*, *in terms of my expectation would be there are things I can do*, *I can make it happen*, *and there are people I can enlist support and allies*. *P8 (Prov)*.

When that sense of agency was lacking, it had the potential to leave the person affected feeling quite disempowered and defeated. P24 (EBE) illustrated this with a recent example of when she was faced with a seemingly insurmountable problem:

*I remember there was a few week back and I was going through hell … all this shit came on top of me*, *like all I wanted to do was get lost in a feeling*, *like just shut down the emotion completely*, *and just get fucking wasted rather than deal with the problem*. *P24 (EBE)*.

#### Life experiences–“I think society is never going to forget” P2 (EBE)

These were significant incidents or events that people who were socially excluded had experienced, and that had adversely affected their lives. They could be seen as markers of disadvantage and vulnerability, with a cumulative effect on the person over time.

Examples included being imprisoned, periods of severe addiction, or having to beg on the streets. Participants described society and services as being quite unforgiving in terms of past behaviour; excluded people were rarely given a chance to reengage. P2 (EBE) described the effects that persistent negative attitudes had on him:

*I often relapsed [on heroin] over it because I would be thinking*, *what’s the point*? *Like the way I looked at it before was*, *I had to change for society not myself*, *trying to fit in*, *but then I learned a valid lesson that*, *hang on–you have to change for yourself first*, *and then try and reintegrate in to society*. *But I have tried this a few times*, *and you always end up [thinking] what’s the point like*? *People are just*, *they have their opinions*, *that’s not going to change … I think society is never going to forget … I suppose when they see me they are saying right there is an ex armed-robber*, *a heroin addict*. *P2 (EBE)*.

Two EBE also took the view that it was up to the affected people themselves to start the work of leaving social exclusion, and that the motivation for doing so had to be personal. It was often felt that when successes did happen, they occurred despite a society and system that was mostly unsupportive. Not surprisingly, the usual result of this was that the more of these past behaviours or challenges a person had, the less likely they were to be allowed to engage with appropriate services.

*The more kind of disadvantaged intersections you have*, *the fewer the resources are there for you because you will not qualify or you will fall through the cracks in a number of ways*, *or your particular needs probably won’t be met*. *P14 (Acad)*.

#### Identity–“There is people like us, and there is people like them, and we are not like them.” P11 (EBE & Acad)

Participants explained that they understood that identity was formed by a combination of elements; where a person had come from, who they had come from (their family and their values), and what they themselves were doing with their lives.

*I am working in the addiction field*, *so I suppose with some of the people that we would work with within their peer groups*, *within their families*, *within their community settings*, *they would have different norms around drug and alcohol use compared to more*, *compared to mainstream society*, *and part of that would be their culture*. *P8 (Prov)*.

Identity for people who were excluded also seemed closely linked to the cumulative unfairness that they, or members of their social group, had suffered in the past. P11 (EBE & Acad) gave the example of a time when she had facilitated the visit of a group of teenage students to the university where she worked. They all lived in disadvantaged areas, and the purpose of the visit was to encourage them to consider applying for entrance to the university. She said that that they seemed very uncomfortable during the visit, and reflected that the likely reason was that “they’ve lived a life where they have been told that they are not good enough.” P11 (EBE & Acad).

There was also discussion that being a member of a group might provide some benefits, even if that group was often stigmatised and ostracised. P1 (CSO) explained, “There is a comfort in everybody being excluded together”. Group identity provided a ‘safety-blanket’ to people in some of the excluded groups, but any questioning of the adverse circumstances they found themselves in may have been considered inadvisable and futile:

*I think it’s quite a difficult thing to get someone to say–Yes*, *I am excluded*, *and here are all the reasons why I am excluded*, *and I want all these different things*, *because it is often outside their remit to be able to achieve those things*. *P19 (Acad & Prov)*.

### Social outcomes

These were seen as the desired results of advancement and leaving social exclusion. They include the ability to care for oneself effectively, being accepted by ‘mainstream’ society and feeling free to participate in that society.

#### Self-care

For a person who had found some degree of stability in their life, being able to look after themselves was seen as an important early stage. Tasks such as planning and preparing meals were a challenge, and P1 (CSO) explained why she felt that was using the example of a past client:

*He was very suspicious of any food he had never tried … I found out that pretty much everything was convenience food in his household*, *but there was so much else going on in his household that I think that they didn’t have the time*, *the energy*, *the money … There was violence in the household*, *addiction in the household*, *extreme poverty in the household*, *mental health issues in the household*. *The fact that they were getting a meal on the table at all was an achievement in a way*. *P1 (CSO)*.

This adversity that particular client had faced on a daily basis was immense, and now an important part of their journey from exclusion was to learn new skills and basic knowledge on everyday tasks.

#### Acceptance–“You are in a limbo, you are in social limbo.” P16 (EBE & Pol)

The idealised pathway for a person to leave exclusion, achieve a degree of stability and eventually be able to participate in society seemed straightforward. In order for that to have had any chance of taking place, however, wider society needed to have made that person feel wanted and welcome. Many participants disagreed with the assumption that mainstream society would automatically welcome people warmly. P5 (EBE & CSO) was adamant that this was a major obstacle to participation for the people that she worked with:

*It’s not internal*, *it’s not inside them—they want to participate*, *it’s the fact that when they try to*, *other people say—Yeah*, *you can do that somewhere else*, *which is the essence of exclusion*. *I come*, *and I asked to come in*, *and you tell me–No*, *go somewhere else and go in*. *Don’t come in here … Go participate in society down the road*. *Yeah there is somebody down there that will have you*, *but I don’t want you participating with me*. *P5 (EBE & CSO)*.

Interviewees explained that in order to progress, some people who were excluded had lost the social connections and supports that sustained them while they were marginalised:

*If you have grown up with a group of people*, *you’ve probably been through a lot of high and lows together*, *and they are very much part of your identity as well*, *and then when you do cross over*, *they don’t come with you*. *Because they don’t speak the same way*, *they don’t look the same way*, *they won’t go to the same places … so then they can exclude you then*. *P11 (EBE & Acad)*.

This meant that the harsh reality for many of those who had made the leap from exclusion was that they ended up feeling unwanted by both the group they had left behind, and the very ‘mainstream’ that they had so often been advised and encouraged to join. This frustrating situation was summarised by P16 (EBE & Pol) who said “So you are in a limbo, you are in social limbo”.

#### Social participation

Participation in wider society was seen as the goal for a person leaving exclusion. This aspirational end-point included being able to engage with others as an equal, and having their voice heard on matters affecting their life and their community.

There were various opinions as to what aspects of life would be important to recognise in terms of that participation:

*It means everything from attending a community meeting*, *to having some kind of say over your neighbourhood*, *to voting*, *having the ability to exercise to express your voice*, *and have your voice be heard and be recognised*, *in any of those kinds of ways … Or not if you don’t want to*, *but to have the freedom to*. *P14 (Acad)*.

“Some people don’t want to be part of what we think” P5 (EBE & CSO).

Differences between what mainstream society presumed was best for people who were excluded, and what the individuals affected actually wanted became obvious during the interviews. P13 (Prov & Pol) explained, “There are many people who have no desire to have full participation in society, and live perfectly good lives” P13 (Prov & Pol). Interviewees proposed a number of possible explanations for that reluctance to try to participate. Some socially excluded people did not want to participate:

*Sometimes we find there are opportunities being provided*, *but people don’t see the value of it*, *or can’t create their own head space to understand why that might be important*. *P15 (Prov)*.

There may have been a tendency to see the decisions made not to participate as short-sighted and irrational, but the reality was that many excluded people did not have any knowledge of what was actually possible for them to do. Even when opportunities to participate did arise, interviewees felt that people who had been excluded, and who had seen the effects of exclusion on people in their social circles, lacked the confidence to go ahead and grasp those chances. There was also the idea that people were being asked to expose themselves, when ultimately their attempt at integration and participation in the mainstream was very likely to fail.

*You are not only asking someone else to participate in society*, *you are asking them to have access to a completely different way of life*, *which they don’t have*, *so they will never feel part of*. *P16 (EBE & Pol)*.

Micro community–“There is a comfort in everybody being excluded together.” P1 (CSO).

The informal support networks that people who were excluded had found and developed over time were seen as important for them in terms of coping and information sharing. The solidarity and empathy that people found in these groupings was often underestimated:

*I think sometimes when you have been excluded yourself you are maybe a bit more conscious about how other people are feeling*, *and you are a bit more tuned in*. *P17 (Acad & Prov)*.

This type of informal group was labelled a “micro community” by P1 (CSO), and she described how they could be beneficial and give a person a sense of identity, but they could also be quite insular and challenging to leave.

*When people move on it threatens the status quo*, *I suppose*. *It rattles it a little bit*, *and others are quick to pull people back*, *or to remind them of their place*, *because it frightens or threatens them in some way … You can see it when people return to education*, *or we can see it sometimes as well when people become peer workers*, *or when people are addressing their addiction issues … I suppose because when everybody is excluded together*, *there is another little micro society created where everyone is included*. *P1 (CSO)*.

This resentment that was sometimes seen when a person attempted to leave exclusion, and therefore leave their ‘micro community’, was an added emotional barrier and disincentive complicating what was often already a very challenging journey.

### Definition & conceptual model

The definition of social exclusion evolved throughout the fieldwork, and the final version was: Social exclusion is the experience of lack of opportunity, or the inability to make use of available opportunities, thereby preventing full participation in society.

**Fig 1:**
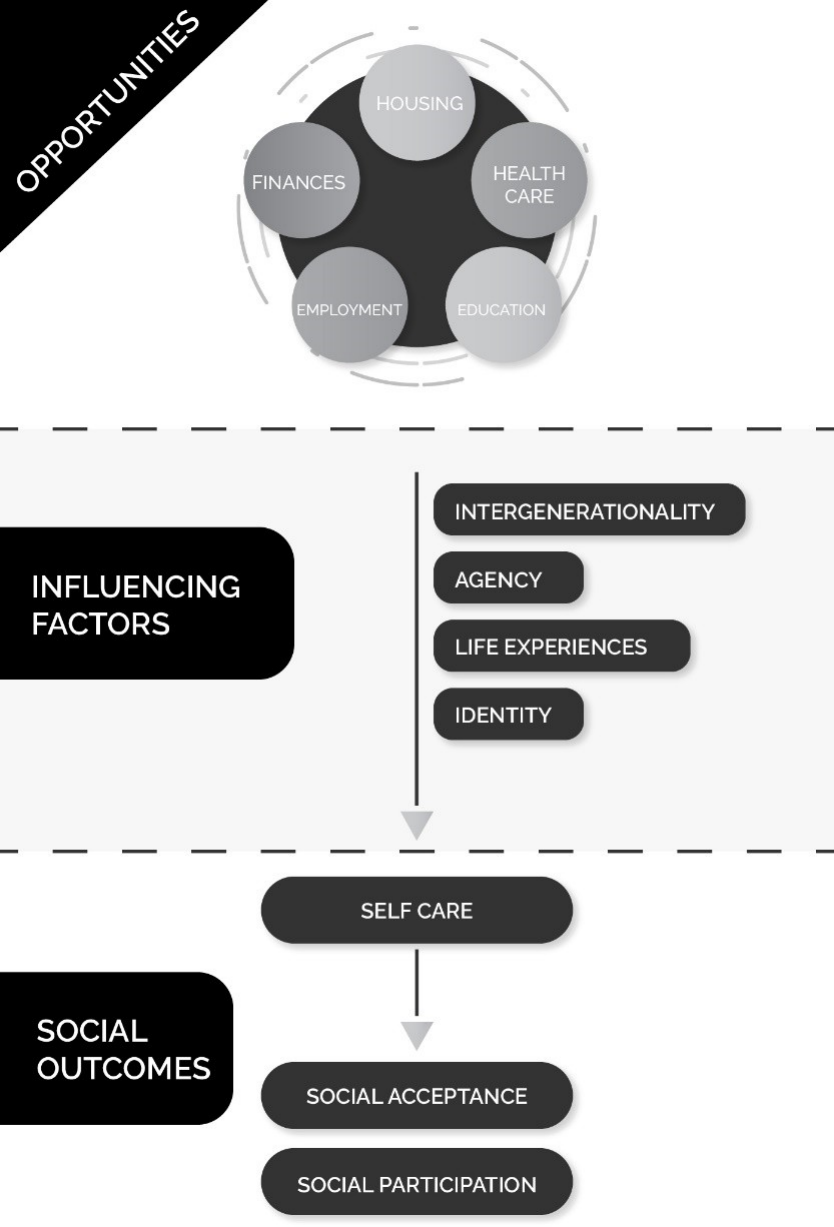
Conceptual model of social exclusion.

The final conceptual model of social exclusion is presented in Fig 1. At the top, we have the Opportunities that need to be accessed relatively freely in order for an individual to successfully leave social exclusion. In the centre, we have Influencing Factors that shape the journey of the individual as they move from exclusion. At the bottom, we then have the Outcomes that result from the Opportunities being moderated by the Influencing Factors.

## Discussion

### Statement of principal findings

Based on our interviews with 24 stakeholders, social exclusion can be defined as “the experience of lack of opportunity, or the inability to make use of available opportunities, thereby preventing full participation in society.” From this, we developed a new model for the concept utilising three elements Opportunities, Influencing factors and Social outcomes. The Opportunities are the fundamental needs that are required to be met for a person to begin to leave social exclusion. The Influencing factors are a mixture of the personal characteristics of an individual and more complex problems such as the intergenerational effects of extreme disadvantage. The Social outcomes included a person being accepted by wider society, and subsequently being able to participate and flourish in that society.

There were some patterns of differences in the responses by research participant groups, particularly with regard to the EBE interviewees. For example, two of the members of this group felt that it was up to a person himself or herself to start the process of moving from social exclusion and beginning to engage more widely. Then later, all of the EBE participants described feeling that exclusion and marginalisation resulted from action or inaction by other actors in the ‘mainstream’ of society. Only three of the non-EBE participants mentioned this point. Elsewhere, both EBE and non-EBE participants highlighted the adverse experiences that socially excluded people had when engaging with health services, and the likely ramifications of this ill-treatment.

### Discussion of findings in relation to the extant literature

The complexity of the concept, and the different meanings ascribed to it by different stakeholders, had been written about in the past [8,14,15]. The challenge of deciding which groups should be considered as excluded had also been discussed, with some bodies attempting to draw up lists as a guide [35]. The way social exclusion was conceptualised by Atkinson [36] and others helped to frame it as a concept that was relative to the ‘mainstream’ where certain privileges existed and norms were seen to occur [8,37]. They also felt that exclusion was an action–an act of commission or omission–by an excluded person themselves or, more commonly, by an outside actor [36]. We found this was a common response from our participants also.

Social exclusion has long been acknowledged as a multidimensional concept, encompassing issues beyond simple income and resource poverty [10,37–39], and this was reflected in the findings from our interviewees. The WHO Social Exclusion Knowledge Network (SEKN) report of 2008 concluded that economic, political, social and cultural aspects of life were all relevant [4]. Our model describes the fundamental ‘Opportunities’ like healthcare and education as being on a par with finance in terms of beginning to tackle social exclusion. There is no doubt that the availability and quality of these five elements dictates greatly whether a person is able to leave exclusion and be healthy [40–42]. These align with the social determinants of health that were set out by Dahlgren and Whitehead [43]. The Opportunities are often the main point of focus for government and CSO initiatives, and work on these may be more common as they are relatively easy to measure and monitor e.g. number of homeless people now housed. Access to these Opportunities is also considered as fundamental rights by some international bodies, placing certain obligations on national governments to provide them [44]. The Opportunities are not the only factors that influenced a person attempting to leave exclusion however, and that was our reason for developing a new conceptual model based on the insights of experts.

We found there were certain Influencing Factors that affected the ability of a person who was socially excluded to progress. Once such consideration was their sense of agency, and in the past authors described how fixed structures and the enormity of the challenges faced by many people who were marginalised trumped their own innate ability to cope and succeed [45–47]. We also found that life experiences such as a history of imprisonment had a negative impact on the individuals’ likelihood of succeeding in trying to stabilise their lives [2,48]. These characteristics were also noted to mark very poor morbidity and mortality outcomes for affected people [2]. Negative societal attitudes and stigma also played a large part in causing these detrimental effects. Lastly, our interviewees saw exclusion that was pernicious across generations of families as mostly insurmountable. Intergenerational disadvantage has been researched and monitored by the European Commission and others as a key indicator of inequity in countries [49,50].

At the level of Social Outcomes there was a noticeable lack of work describing the experiences of those who had attempted to leave social exclusion, and had either succeeded or failed at doing so. There had been research carried out with those who were excluded, and it documented that exclusion [51,52], but we could find very little on the attempts that people made to overcome this exclusion. This is despite the SEKN report recommending that recording relevant “stories” was as important as collecting quantitative data [4]. Our participants noted that being able to overcome exclusion and flourish in the ‘mainstream’ was a difficult and rare occurrence. We also found that it was wrong to presume that every individual had the desire to function and conform as part of that idealised ‘mainstream’. The negativity and difficult experiences of some who had made the leap from exclusion may have acted as a disincentive to others who considered following them.

### Strengths and weaknesses of the research

This research included the voices of a diverse range of key informants. One quarter of the participants were, or had been, EBE and this was a key strength. We also had policy makers and service planners take part, and this contributed to a comprehensive overview of the concept. We sought to ensure the reliability and validity of the work by using respondent validation, highlighting negative accounts in the data, providing high quality quotes as evidence and cross-checking coding within the research team [53]. Another strength was that the lead researcher had been providing healthcare for socially excluded people for years. As a result, he knew some participants and gatekeepers, and he understood the context prior to beginning this research. The limitations of this research could include the point that the participant sample is not representative, but we utilised maximum variation sampling to attempt to reduce this risk.

### Practice, research and policy implications

In terms of suggestions for practice, the acknowledgement and routine measurement of the social exclusion status of people who engage with primary care and other services would be an important first step. Primary healthcare settings are currently underutilised in terms of recognising the potential exclusion of some the many people attending on a daily basis worldwide. Medical services obviously cannot provide solutions to all material and psychosocial needs of these patients, but they can be extremely effective at signposting appropriate services and supporting patients while they engage [54]. To do this, however, primary healthcare services need to firstly understand the needs of socially excluded people they serve, and then adapt themselves to attempt to support those needs [9,10,55].

The framework developed here can contribute to a better understanding of the concept of social exclusion and how it may be further researched. The burgeoning Inclusion Health movement will hopefully go some way towards improving the level of critical thinking on this, but more can be done to prevent the structures of the health system remaining as inflexible and daunting as they have mostly been heretofore. In terms of further research, the difficult journey faced by those who are attempting to leave exclusion, and those who would consider themselves to have been successful, would be very important to explore further.

As we have already stated, much of the policy focus in terms of mitigating social exclusion currently takes place at the Opportunities level. There is no doubt that having a basic level of these Opportunities is crucial for an individual to be able to leave exclusion, but these need to be complemented by work at the level of the Influencing factors and beyond. Actions that could be taken on these factors in order to support people to leave social exclusion include working to disrupt vulnerability that is handed down through generations, supporting initiatives that assist people with adverse life experiences to assimilate and working to educate wider society on the damage done by stigma and intolerance.

## Conclusions

Social exclusion was defined as “the experience of lack of opportunity, or the inability to make use of available opportunities, thereby preventing full participation in society.” We developed a new conceptualisation of the concept with three levels: Opportunities, Influencing factors and Social outcomes. Opportunities are fundamental elements that are required to be in place in order for a person to leave social exclusion. Influencing factors are a mixture of the personal characteristics of an individual and more complex issues such as the intergenerational perpetuation of extreme disadvantage. Social outcomes including being accepted by wider society, and being able to participate, are seen as aspirational for the majority of socially excluded people.

## Supporting information

S1 File (DOCX)
