# Supplementary material for: “There is people like us and there is people like them, and we are not like them.” Understating social exclusion – a qualitative study
Source: PLoS One. 2021 Jun 22;16(6):e0253575. doi: 10.1371/journal.pone.0253575 (PMC8219157; doi:10.1371/journal.pone.0253575)
Supplement: S1 File — (DOCX) [file pone.0253575.s001.docx]

## Appendix 1

Interview Topic Guide:

**Social exclusion is a term that is used by many people in many different settings, sometimes with different meanings. I’d like to get your views and ideas about what social exclusion means to you, and what you think it is like to be socially exuded in our society today.**

1. How would you define “social exclusion” in your own words? What does it mean to you?
2. What does it mean to you to be able to fully participate in our society?
3. What kind of things might make it difficult for people to fully participate in our society?
4. What kind of things might mean someone is vulnerable to social exclusion?
5. Who is affected by social exclusion in Ireland?
6. What do you think the effects of social exclusion are?
7. Some say that social exclusion is related to health and wellbeing, would you agree?
8. Can a person go from social exclusion to social inclusion (SI), and how can they do that?
9. Who ‘works’ on social exclusion in Ireland?
10. Who should be concerned with social exclusion in Ireland, and why?
11. Some say that there is a relationship between social exclusion and Primary Health Care, what do you think about that?

Experiences of social exclusion

1. Do you personally know anyone who has any experience of being socially excluded? Can you tell me about their experience?
2. Do you have any experience of being socially excluded yourself?
3. Could you tell me about your (his/her) experience.
4. Can you give me an example of the last time you (or that person) felt excluded?
5. Can you tell me about your (his/her) journey from SE to where you are (he/she is) now, and explain what helped you (he/she) on that journey?
